# Human tissue models in cancer research: looking beyond the mouse

**DOI:** 10.1242/dmm.031260

**Published:** 2017-08-01

**Authors:** Samuel J. Jackson, Gareth J. Thomas

**Affiliations:** 1 National Centre for the Replacement, Refinement and Reduction of Animals in Research, Gibbs Building, 215 Euston Road, London NW1 2BE, UK; 2 Cancer Sciences Unit, Faculty of Medicine, University of Southampton, Somers Building, MP 824 Tremona Road, Southampton SO16 6YD, UK

## Abstract

Mouse models, including patient-derived xenograft mice, are widely used to address questions in cancer research. However, there are documented flaws in these models that can result in the misrepresentation of human tumour biology and limit the suitability of the model for translational research. A coordinated effort to promote the more widespread development and use of ‘non-animal human tissue’ models could provide a clinically relevant platform for many cancer studies, maximising the opportunities presented by human tissue resources such as biobanks. A number of key factors limit the wide adoption of non-animal human tissue models in cancer research, including deficiencies in the infrastructure and the technical tools required to collect, transport, store and maintain human tissue for lab use. Another obstacle is the long-standing cultural reliance on animal models, which can make researchers resistant to change, often because of concerns about historical data compatibility and losing ground in a competitive environment while new approaches are embedded in lab practice. There are a wide range of initiatives that aim to address these issues by facilitating data sharing and promoting collaborations between organisations and researchers who work with human tissue. The importance of coordinating biobanks and introducing quality standards is gaining momentum. There is an exciting opportunity to transform cancer drug discovery by optimising the use of human tissue and reducing the reliance on potentially less predictive animal models.

## Abstract (precis)

**Summary:** Samuel Jackson and Gareth Thomas discuss the limitations of patient-derived xenograft mouse models and highlight initiatives to maximise the use of human tissue in cancer research, with the goal of improving translation and reducing animal experimentation.

## Introduction

In 2015 in Great Britain, cancer research accounted for nearly 200,000 experimental procedures on animals – around 5% of all procedures carried out during that year (www.gov.uk/government/statistics/statistics-of-scientific-procedures-on-living-animals-great-britain-2015). This high level of research reflects the increasing societal burden of cancer and the substantial level of funding this area attracts. Research into the mechanisms of cancer pathology and cancer drug development have relied heavily for many years on genetically modified, cell line xenograft and more recently, patient-derived xenograft (PDX) mouse models, which have produced key mechanistic insights into cancer biology (Gopinathan et al., 2015; Holen et al., 2017). The high level of molecular, genetic, cellular and physiological conservation between vertebrates have made mice attractive for studying systemic diseases such as cancer. However, the translational relevance and predictivity of mouse models used for testing cancer therapeutics has been questioned (Moreno and Pearson, 2013). There are emerging technologies and approaches that have the potential to increase the relevance of preclinical models to human disease. The greater use of human tissue presents one such opportunity.

Human tissue has been used for many years to study cancer. Nevertheless, access to high-quality tissue remains problematic, with a lack of coordination at a national level. In the UK, collaborations tend to be based on personal connections between clinical and research staff, or local biobanks that provide tissue to a small network of researchers. It is clear that the provision of human tissue in research settings would benefit from more widespread integration between these parties. Here, we highlight some key limitations of PDX mouse models for cancer research, and illustrate alternative uses for human tumour and normal tissue that reduce reliance on animal models and have the potential to accelerate the development of cancer therapeutics.

## The problem with PDX mice

In recent years, there has been a large rise in the use of PDX mice in cancer research. Many studies demonstrating the successful translation from PDX mouse to clinical drug response have been published, underlining the current status of the PDX as a gold standard model (Julien et al., 2012; Weroha et al., 2014). The increase in outsourcing of experiments from pharmaceutical companies to contract research organisations has driven the expansion of commercial PDX colonies, and concomitantly the range of cancer types and subtypes now available has widened. The use of PDX models in academic research has also expanded, with the number of publications from academic research groups increasing exponentially since the start of the millennium. Biologically, the implanted tissue largely retains the heterogeneity and architecture of the original tumour, and the size of a subcutaneous tumour can be easily assessed in response to a treatment. Importantly, PDX implants can be passaged into another group of animals, allowing maintenance of the tissue for several ‘generations’ (Pompili et al., 2016).

However, the success rate of engraftment of human tumour tissue into the mouse is variable. Some tumours are easy to cultivate, while others cannot be grown, resulting in selective bias at the very earliest stage of PDX model development. This means that some human cancer subtypes are not represented in PDX mouse collections. Often more aggressive tumours with high proliferation rates are easier to propagate, resulting in a population of PDX mouse models skewed towards these phenotypes (Zhao et al., 2012). Past issues with clonal selection and genetic drift have been largely overcome by the use of low-passage number grafts (Hollingshead et al., 2014). However, this means that primary engraftment is carried out more often, requiring more animals as well as increased amounts of human tissue, which may be wasted if tumours fail to engraft. Tumour tissue may arise from a range of anatomical sites in humans, but is almost always implanted under the skin in the mouse, and not in the orthotopic site. This exacerbates the lack of relevant tissue-specific support for the implanted tumour, and contributes to different responses in mouse and human (Junttila and de Sauvage, 2013). When engraftment is successful, the stromal component of the tissue is often remodelled and replaced with mouse-derived stroma. Mouse and human stromal fibroblasts have been shown to react differently to treatments, meaning that some PDX mice are not reliable therapeutic test-beds (Kalluri, 2016).

There is also a potential welfare burden on mice used for PDX studies. Implanted subcutaneous tumour tissue needs to grow to a certain size before removal is practical, or to see a statistically and biologically relevant pharmacological effect on tumour size (Workman et al., 2010). The graft itself may cause pain or distress, and animals or experiments may need to be terminated early due to grafts becoming ulcerated. Analgesia may not be given due to concerns about confounding experimental results (Page et al., 2001; Sasamura et al., 2002) and there have been few studies assessing objective indicators of pain in experimental cancer models. Given these issues and the scientific limitations, it is important to assess whether PDX represents the best use of human tissue, and whether alternative approaches can provide more relevant information without the use of animals.

## A better use of human tissue for cancer research?

Non-animal human tissue models can overcome some of the limitations of PDX mice, in part because there are many tissue culture methods available. Human tissue- or cell-based models have increased in complexity, and output parameters have diversified beyond simple measurements of proliferation, invasion and cytotoxicity to include critical steps in the metastatic cascade, angiogenesis and tumour immunity. Advances in 3D cell culture, bioengineering and microtechnologies have contributed to the rapid development of novel *in vitro* models that incorporate multiple cell types, extracellular matrix proteins and soluble factors that constitute the tumour microenvironment (Albritton and Miller, 2017). Human tissue models can be divided into two general categories: those that preserve the architecture of the original tissue (‘top down’), and those that recreate the architecture from scratch (‘bottom up’). The use of tumour tissue *in vitro* has gained popularity through the availability of new technologies, including microfluidics, and developments in outcome measurements, such as tissue imaging. These top-down methods preserve the structure, mutational load and heterogeneity of the original patient tumour, and include tumour slice culture (Davies et al., 2015) and explant microtissues (Villasante and Vunjak-Novakovic, 2015).

Recent advances in human stem cell technologies have expanded the definition of human tissue to include induced pluripotent stem cells (iPSCs). These cells have made the bottom-up construction of tissue models feasible, and provide a scalable source of material of human origin. iPSC-based models have gained in popularity as the technology matures, and approaches including organoids (Di et al., 2014) and decellularised extracellular matrix ‘scaffolds’ (Chen et al., 2016) have been applied to recreate tumour architecture in 3D. Cross-disciplinary research and collaboration has led to engineering solutions to tissue maintenance, such as microphysiological systems (Esch et al., 2015). Both top-down and bottom-up paradigms have provided significant technological innovations; however, the challenge remains as to how these will be best employed or optimised to address specific research questions.

When tumour tissue is cultured *in vitro*, the microenvironment can be tuned to encourage growth of the implanted material (Asghar et al., 2015) and more options are available for manipulation and analysis than in mice. For example, single-cell imaging (Antfolk et al., 2017) and real-time biomarker monitoring (Washburn et al., 2016) can provide data on tumour response to a compound at a high granularity and throughput (Horvath et al., 2016); such high-sensitivity analyses may enable shorter experiment times and yield more detailed phenotypic information than the equivalent mouse experiment (Sandercock et al., 2015). The cell- and tissue-based techniques described, together with advances in multi-organ chip technologies, have the potential to replicate the systemic nature of vertebrate physiology *in vitro* (Portillo-Lara and Annabi, 2016).

While many of the principles of immunotherapy have been informed by mouse models (Budhu et al., 2014), cancer drug developers have an increasing need for more advanced models that faithfully recapitulate the human immune system. This need has led to the development of mice with humanised immune cell lineages. These models are expensive to produce and immune cells often exhibit different responses to immune mediators when compared with human (Zitvogel et al., 2016). Increasingly, non-animal human tissue models have the potential to inform the development of immunotherapeutics, because 3D tissue constructs can be reconstituted with human immune cells with an appropriate phenotype (Linde et al., 2012). It is thus likely that such tissue-based models will inform future cancer immunotherapy strategies.

## Overcoming the blocks to increased uptake of human tissue models

Significant obstacles exist that limit the widespread implementation of non-animal human tissue models in cancer research laboratories. These were discussed at a recent workshop held by the National Centre for the Replacement Refinement and Reduction of Animals in Research (NC3Rs; www.nc3rs.org.uk). The most consistently cited barrier to uptake by delegates at the workshop was a lack of access to high-quality tissue with associated clinical metadata. In order to meet this demand, the biobanking infrastructure supporting the collection, transport and storage of human tissue and cells needs to be developed and/or improved.

Historically, biobanking infrastructure in the UK has received poor support and lacks coordination. However, there are signs that attitudes to the systematic collection of human tissues, particularly in the context of clinical trials or large observational studies where tissues are linked to detailed clinical outcome data, are changing. Recent initiatives include TRACERx (www.cruklungcentre.org/Research/TRACERx) and Precision Panc (www.precisionpanc.org/), which are large-scale programmes dedicated to the collection and genetic analysis of lung and pancreatic cancers respectively. The knowledge derived from these initiatives highlights the importance of standardising the nomenclature and ontology used for biobanking; benchmarking tissue quality, including methods of tissue collection, transport, preservation and analysis; and obtaining consent and procedures to provide relevant and sufficient metadata. Taking account of these principles and standardising practice will be essential to expedite the use of non-animal human tissue models.

Technical deficiencies also hamper the implementation of human tissue models. For example, tissue degradation or changes in protein stability and cellular homeostasis after removal can affect tissue quality (Grizzle et al., 2010), meaning that patient-to-incubator delay times should be kept to a minimum. The development of tools to aid the collection, transport, storage or maintenance of fresh human tissue will be vital to enable use in a research setting. In particular, the development of products or techniques to preserve tissue in a viable state for longer after removal would greatly improve the tissue supply chain.

It is important to recognise that researchers can be reluctant to invest time and money in implementing a new technique, or to replace an animal model that has served as the basis of their research for many years. A number of potential reasons for this were discussed at the NC3Rs workshop. There may be concerns about a lack of historic data comparability, or invalidating past results. Setting up a new model can require additional technical expertise or development of new infrastructure. Referees are familiar with data from the ‘gold standard’ animal models, and may request additional *in vivo* data to be generated to support *in vitro* findings. These factors can delay publication in a highly competitive research environment and result in a lack of motivation to change models.

## The alliance of tissue culture and animal models

The ethical and cost implications of carrying out large-scale drug screening or complex mechanistic studies in PDX mice can make this approach undesirable or unfeasible. One alternative strategy is to test a drug or hypothesis in tissue culture models derived from patient samples prior to smaller-scale studies in mice (Bruna et al., 2016; Sandercock et al., 2015). *In vitro* approaches can help define the best downstream use of animal models while reducing animal numbers and building confidence in human tissue modelling. It is worth noting that alternative animal-based methods can be used to reduce rodent numbers and cost for drug or hypothesis testing. This includes the xenotransplantation of human cancer tissue into zebrafish embryos, which have been used to study cancer, including breast cancer bone metastasis, and can provide an alternative or adjunct to mouse PDX models or tissue culture (Feng and Martin, 2015; Mercatali et al., 2016).

To prove the value of human tissue models a comparison is needed, both against the human disease to demonstrate translational value, and against the animal model to provide a basis by which to compare historic data (while avoiding additional animal experiments). However, traditional funding schemes do not usually support comparative research of this kind, and often relevant human or animal model data is held confidentially by companies. Sharing of experiences, expertise and experimental results between groups is a proven method of qualification of models during and after development, and provides an evidence base to inspire confidence in the modelling approach. To facilitate this, it will be important to raise awareness of the potential of human tissue techniques amongst researchers and clinicians, and engage these communities to enable them to work together towards this common goal.

The NC3Rs has held a series of workshops to share experiences and to examine how human tissue use can be supported in a range of areas, including cancer. Through these and associated initiatives such as the Human Tissue Hub online resource (www.nc3rs.org.uk/increasing-human-tissue-use) and the collation of case studies and best practice, we aim to centralise information about human tissue modelling and provide a platform for dissemination of this information.

### Conclusions

Now, unlike any time previously, technologies are emerging that make alternatives to animal use a reality. Modern translational cancer research could benefit from the pursuit of human tissue-based models. However, creating an environment where these models can be used to replace some PDX studies will be challenging and there is a need for stakeholders including research funders to explore how this can best be facilitated. Widespread application of human tissue models will require investment in infrastructure and training, and the implementation of strategies to ‘de-risk’ the adoption of new models, including data comparison and sharing. The challenge for the future of the field will be how to provide proof-of-concept and validity of these paradigms, and expedite incorporation of new models into the research process.
